# A Unified Account of Perceptual Layering and Surface Appearance in Terms of Gamut Relativity

**DOI:** 10.1371/journal.pone.0113159

**Published:** 2014-11-17

**Authors:** Tony Vladusich, Mark D. McDonnell

**Affiliations:** 1 Institute for Telecommunications Research, University of South Australia, Mawson Lakes, 5095, Australia; 2 Center for Computational Neuroscience and Neural Technology, Boston University, Boston, MA, United States of America; University of Ulm, Germany

## Abstract

When we look at the world—or a graphical depiction of the world—we perceive surface materials (e.g. a ceramic black and white checkerboard) independently of variations in illumination (e.g. shading or shadow) and atmospheric media (e.g. clouds or smoke). Such percepts are partly based on the way physical surfaces and media reflect and transmit light and partly on the way the human visual system processes the complex patterns of light reaching the eye. One way to understand how these percepts arise is to assume that the visual system parses patterns of light into layered perceptual representations of surfaces, illumination and atmospheric media, one seen through another. Despite a great deal of previous experimental and modelling work on layered representation, however, a unified computational model of key perceptual demonstrations is still lacking. Here we present the first general computational model of perceptual layering and surface appearance—based on a boarder theoretical framework called gamut relativity—that is consistent with these demonstrations. The model (a) qualitatively explains striking effects of perceptual transparency, figure-ground separation and lightness, (b) quantitatively accounts for the role of stimulus- and task-driven constraints on perceptual matching performance, and (c) unifies two prominent theoretical frameworks for understanding surface appearance. The model thereby provides novel insights into the remarkable capacity of the human visual system to represent and identify surface materials, illumination and atmospheric media, which can be exploited in computer graphics applications.

## Introduction

The human visual system manifests the remarkable capacity to identify surface materials from the complex patterns of light reaching the eye [1], [2]. This capacity is exploited in the computer graphics industry to create convincing renderings of surface materials based on physical models of ‘light transport’ [3]–[5]. The problem of understanding how the visual system represents surface materials (e.g. ceramic tiles or human skin), and related visual properties of illumination (e.g. shadows, shading and highlights) and atmospheric media (e.g. clouds, fog and smoke), is thus of immense practical importance in the field of computer graphics.

**Figure 1:**
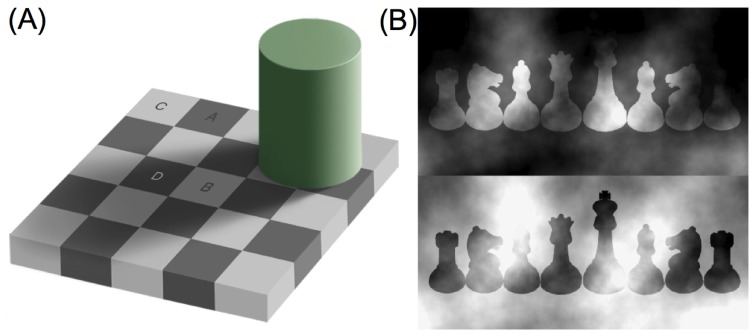
Two dramatic effects of perceptual layering and surface appearance. (A) Adelson checkerboard image [1] adapted from http://web.mit.edu/persci/people/adelson/checkershadow_illusion.html under the Creative Commons Attribution License: Checks labelled A and B (depicted as appearing in bright and dim illumination) have the same point-to-point luminance but check B appears light gray and check A dark gray. Checks B and D are seen through a ‘transparent shadow layer’, whereas checks A and C are seen in ‘plain view’ (without an accompanying transparent layer). Variations in illumination intensity level produce multiplicative changes in the luminance values depicted as being reflected from the checks in bright and dim illumination. (B) Anderson-Winawer effect reprinted from [12]: Chess pieces in the upper and lower rows have the same point-to-point luminance but appear white and black, respectively. The white pieces are seen through a blackish transparent ‘atmosphere’ whose transparency varies across space, while the black pieces are seen through a transparent whitish atmosphere. Variations in atmospheric transmittance levels produce additive changes in the luminance values depicted as being reflected from the black and white chess pieces. This article develops a model that aims to quantitatively predict surface lightness through transparent layers, irrespective of the physical source of the transparent layer.

Models of physical light transport attempt to capture the immensely complicated ways in which physical surfaces and atmospheric media reflect, refract, scatter and transmit light [3]–[5]. The net result is that the light patterns reaching the eye from a rendered image consist of a mixture of physically modelled causes. Light ‘reflected’ from a rendered transparent surface using a standard *α*-blending model, for example, is combined with light ‘transmitted’ through the surface from the background [6]. Thus, even simple diffuse shading and/or blending models produce images that the human visual system parses into layered perceptual representations, one seen through another, as illustrated by the striking perceptual effects shown in Fig. 1. How the human visual system parses such images into separate material, illumination and atmospheric layers remains a challenging problem in both human vision science and computer vision science.

In this article, we study the ‘mid-level’ computations that give rise to perceptual layering and related surface appearance properties, such as lightness and transparency, in images generated using simple diffuse shading and *α*-blending models [1], [6]–[34]. Such mid-level computations evolved to process light associated with real physical sources, but in this article we will consider the more circumscribed issue of how the visual system represents surface materials, illumination and atmospheric media associated with graphically rendered physical sources. In this respect, the focus of this article will be the analysis of rendered images that elicit decomposition into surface and shadow/atmospheric layers (perceptual layering), rather than real physical scenes, which are known to sometimes elicit different perceptual interpretations when compared to rendered images [35]–[37]. We will also leave for future work the complex issue of how to model surface appearance in images that are difficult to interpret in terms of globally consistent perceptual layers, such as images containing certain types of gradients [37]–[39].

The perceptual effects shown in Fig. 1 are known as the Adelson checkerboard effect (Fig. 1A) [1] and the Anderson-Winawer effect (Fig. 1B) [12], [13], respectively. In both effects, figure regions having the same point-to-point luminance distribution are perceived as having very different lightness due to variations in the surrounding ‘ground’ regions, which induce the impression of surfaces seen through different types of ‘overlays’. In the Adelson checkerboard effect (Fig. 1A), grayish background checks are seen through a shadow cast over part of the display, whereas in the Anderson-Winawer effect (Fig. 1B), blackish or whitish chess pieces are seen through a cloud bank or wall of smoke that varies in its transparency at different points.

The demonstrations shown in Fig. 1 raise a number of important modelling challenges. First and foremost, a computational model is needed to explain how the human visual system represents different sources of physical variation—such as surfaces, illumination and atmospheric media—in terms of layered perceptual representations. Although much experimental and modelling work has been done on the topic of layered representations, and their relevance to surface material perception, a unified computational model of key perceptual layering effects is still lacking [1], [6]–[18], [26]–[34], [39]. Second, the model must address the difficulty that variations in illumination intensity, such as shadows and shading, are associated with multiplicative changes in registered luminance, whereas variations in the transmittance of physical surfaces and atmospheric media are associated with additive changes in luminance [8], [12], [13], [24], [39]. Third, the model needs to incorporate an understanding of the manner in which the visual system represents the transparency of rendered physical surfaces and atmospheric media [6], [9]–[13], [15]–[18], [29], [32]. Fourth, the problem of separating an image region into perceptual layers is closely related to the problem of determining which surface regions appear in plain view and which appear through the transparent overlay, and thus requires an analysis in terms of figure-ground relationships [12], [13], [40].

Demonstrations of the sort illustrated in Fig. 1 also indirectly highlight the importance of considering stimulus- and task-driven constraints on surface appearance [37], [39], [41]–[50]. This is because stimulus- and task-driven constraints play a critical role in determining whether the visual system computes one or more perceptual layers [12], [13]. In this article, we link stimulus- and task-driven constraints on the computation of perceptual layers to key perceptual matching data on the role of stimulus- and task-driven constraints on brightness (luminance) and lightness (reflectance) perception, respectively [25], [30], [41]–[51]. Of particular importance is the problem of teasing apart the complex relationship between the computational processes underlying different aspects of brightness and lightness perception. It is well known, for example, that human subjects adopt different strategies to perform matching tasks (e.g. brightness and lightness) under different stimulus conditions [41]–[45], [48], [49].

The following section of the article briefly reviews several key theoretical concepts underlying our model. The “Model” section then provides the detailed descriptions of empirical studies, mathematical equations, and computational specifications that are needed to explain perceptual data concerning the demonstrations shown in Fig. 1. The “Results” section provides conceptual analyses and computer simulations of the model under various stimulus- and task-driven constraints, demonstrating the model's capacity to quantitatively predict perceptual data. The “Discussion” section briefly explores some broader implications of the theoretical framework on which the current model is based.

## A Brief Review of Gamut Relativity

The model we present is based on a recently introduced theoretical framework known as *gamut relativity* [52]. The interested reader can find detailed background information in several recent publications [52]–[57].

### Blackness and whiteness are orthogonal dimensions

**Figure 2:**
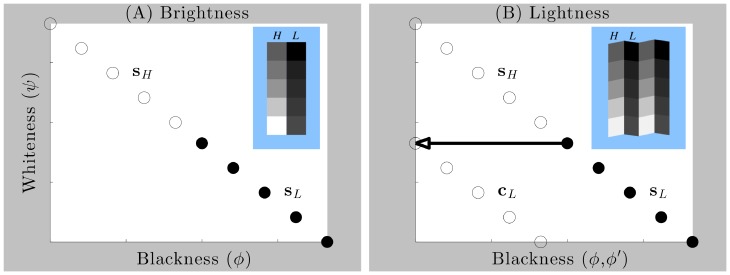
The representation of brightness and lightness in gamut relativity. (A) Surface regions represented under the assumption of a single illumination level and a planar arrangement of surfaces, such as co-ordinates 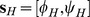 and 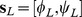, fall on a negatively sloped ‘gamut’ line in blackness-whiteness space, where 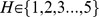 and 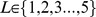 denote the columns of relatively higher and lower luminance squares depicted in the insets, respectively. (B) Surface regions represented under the assumption of two different illumination intensity levels and a corrugated arrangement of surfaces, such as co-ordinates 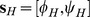 and 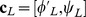, fall on two different gamut lines (termed standard and comparison, respectively). The inset figures in (A) and (B) perceptually illustrate how identical sets of luminance values can be parsed according to the assumptions of uniform or variable illumination levels, respectively. In (A), pictorial image cues indicate that the bright and dark columns of squares (sets 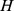 and 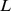) lie in the same depth plane, favoring the assumption of uniform illumination over all squares [25], [89], [90]. Horizontal pairs of squares are thus mapped to different blackness co-ordinates, 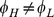. As blackness co-ordinates constitute the computational correlate of diffuse reflectance in gamut relativity, squares in sets 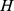 and 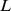 appear to have different diffuse reflectance. In (B), the same sets of luminance values shown in the two columns in (A) are now pictorially depicted to lie in different depth planes (the repetition of rows here enhances this depiction), favoring the assumption of variable illumination [8], [25], [85], [89], [90]. Horizontal pairs of squares in this arrangement are mapped to the same blackness co-ordinates, 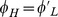, and thus appear to have the same diffuse reflectance. The horizontal vector depicts the shift of points from standard to comparison gamuts, which compensates for the presumptive illumination difference between sets 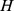 and 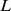. Figure modified with permission from [56].

Our model explains how the visual system represents surfaces independently of variations in either illumination intensity (e.g. shadows; Fig. 1A) or atmospheric transmittance (e.g. clouds; Fig. 1B) in terms of computations performed in a *blackness-whiteness coordinate system* (Fig. 2). Roughly speaking, the whiteness coordinate value (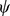) increases with both increasing luminance and positive contrast magnitude, whereas the blackness coordinate value (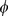) increases with decreasing luminance and increasing negative contrast magnitude. Blackness and whiteness are conceptualised as orthogonal dimensions of a two-dimensional (2-D) perceptual space [52]–[54] that can be ‘sliced’ in different ways, depending on stimulus conditions and task constraints.

### Brightness and lightness are relative concepts

When illumination is perceived as uniform across a scene or object, luminance values corresponding to surfaces with different physical reflectance values are mapped to points falling on a single straight line (‘slice’) in blackness-whiteness space, termed the standard gamut line (Fig. 2A). We associate this mapping with the notion of ‘brightness’ perception. When illumination is perceived as non-uniform, by contrast, luminance values corresponding to different physical surfaces in bright illumination are mapped to points falling on the standard gamut line, whereas luminance values corresponding to different physical surfaces in dark illumination are mapped to points falling on one or more comparison gamut lines (Fig. 2B). The shifting of points from the standard to the comparison gamut line thus compensates for the difference in illumination levels between bright and dark. Vertically aligned points sharing the same blackness coordinates but falling on different gamut lines thus correspond to surfaces with the same physical reflectance [52]. We associate this mapping with the notion of ‘lightness’ perception.

In our model, then, it is the *relationships* between points lying on the standard gamut line—or between points lying on the standard and comparison gamut lines—that determine the properties characterising what we know as ‘brightness’ (Fig. 2A) and ‘lightness’ (Fig. 2B) perception, respectively. This emphasis on relationships between points lying on gamut lines is also the origin of the term ‘gamut relativity’.

### The reflectance-to-lightness mapping is relative

This distinction between our model and alternative models has a number of important correlates. Firstly, as blackness-whiteness space is two-dimensional, invariance along one dimension obviously does not imply invariance along the other dimension, meaning that surfaces sharing the same blackness coordinates needn’t appear *identical*. Secondly, blackness coordinates vary from zero to some arbitrary upper bound, so do not themselves represent a range of ‘lightness values’ varying from black to white. Thirdly, different gamut lines represent different unique slices of blackness-whiteness space, with each line bookended by *different shades of black and white*. There thus exists no absolute mapping from reflectance to gray shades in gamut relativity—in the sense of an absolute scale of lightness values—and this proposal is consistent with a great deal of perceptual data that cannot be explained by classical approaches [52]. In short, our model underlies a more subtle relative account of the reflectance-to-lightness mapping than the classical absolute (scalar) reflectance-to-lightness mapping [25].

### Luminance and contrast sum vectorially to facilitate figure-ground perception

The proposed illumination-shift process described above requires the visual system to compute the local luminance associated with each surface region [52] (and be capable of discriminating illumination edges from reflectance edges [22], [25], [46], [58]). Another key idea in gamut relativity, then, is that luminance, in addition to contrast, plays a central role in determining surface appearance. This idea—as an anonymous reviewer of this article states—“flies against what we currently know about vision...current wisdom is that vision is not sensitive to luminance, only contrast.” Our previous modelling successes—combined with the new analyses presented in this article—suggest that a modest revision to this conventional wisdom may be in order. In particular, we have previously shown how luminance and contrast can be represented as vectors that sum in blackness-whiteness space [54]; the proposed summation of luminance and contrast is consistent with recently reported cortical physiological data [59], [60]. Here we show how this vector summation can facilitate perceptual layering and figure-ground perception by operating asymmetrically on figure and ground image regions (see Results).

### Luminance is also important for ambient illumination perception

The sensitivity to luminance in our model also overcomes a key limitation of approaches based solely on contrast [58], [61]; namely, how is it that we readily perceive variations in ambient (global) illumination? Psychophysical experiments showing that humans can distinguish light levels in *Ganzfeld* stimuli (i.e. containing no contrast) testify to the sensitivity of the visual system to global luminance [62], [63]. Many classical and recent physiological studies [64]–[75] have, furthermore, revealed that both local and global luminance signals are present at early levels of both the cat and primate visual systems—although luminance signals are typically weaker than contrast signals, as documented in the classical early physiological studies of [76], [77]—and recent studies have emphasised the functional importance of these signals in shaping the ON and OFF responses of visual cortical neurons [59], [60], [70], [73], [74], [78]–[82]. Our model emphasises and interprets the available evidence concerning physiological luminance and contrast coding in terms of the relative contributions of these signals to surface appearance; see [52], [54] for further discussion.

### Gamut relativity is versatile and generalises effectively

A significant conceptual advantage of the gamut relativity framework is its ability to account for a wide range of perceptual phenomena in a parsimonious manner [6], [8]–[13], [22], [23], [25], [29], [32], [83]. In addition to specifying brightness and lightness, for example, gamut relativity can also be used to specify the transparency level of a partially transmissive foreground surface or medium. The key idea is that the transparency level of the foreground layer is given by the distance between the standard and comparison gamut lines [55]. The equations of gamut relativity quantitatively explain some puzzling aspects of key demonstrations in classical studies of transparency perception [55], such as the observation that whitish transparent layers appear more opaque than blackish layers with the same physical transmittance [11], [32]. This observation has proven difficult to explain in terms of classical transparency models [6]. Gamut relativity has also been extended to the domain of specularly reflecting surfaces to provide a unified account of layered perceptual representation in lightness and gloss perception [56].

### Existing gamut relativity models need to be combined

The model presented in this article represents a unification of several previously published gamut relativity models that have dealt separately with aspects of brightness/lightness perception [52], [54], lightness/transparency perception [55] and lightness/gloss perception [56], respectively. The latter two studies incorporated only luminance signals in the implemented models (e.g. the model depicted in Fig. 2). Here we show how these previous models can be combined—in a way that incorporates both luminance and contrast—in order to predict data on surface lightness perception through generically defined transparent overlays, whether they be associated with cast shadows, surface shading, atmospheric media or transmissive physical filters. The model goes beyond previous work by (a) qualitatively explaining some striking effects of perceptual transparency, figure-ground separation and lightness perception, (b) quantitatively accounting for the role of stimulus- and task-driven constraints on brightness/lightness matching performance, and (c) unifying two prominent theoretical frameworks for understanding surface appearance (see Discussion). The model thus provides the first quantitative account of perceptual data on the role of stimulus- and task-driven factors in brightness and lightness perception, in terms of a general theory of perceptual layering and surface appearance [25], [30], [41]–[52].

## Materials and Methods

### Perceptual data to be modelled

To motivate the computational modelling, consider the Adelson checkerboard effect (Fig. 1A), which is itself the product of two subtle image manipulations. Firstly, checks A and B—which have the same luminance but whose gray shades appear quite different—are seen against surrounding checks that themselves differ in luminance: check A is seen against checks of higher luminance (labeled check C), while check B is seen against checks of lower luminance (labeled check D). This contextual difference induces the perceptual effect known as *simultaneous contrast* [25], whereby a target seen against a background of relatively higher luminance will appear relatively blacker than a target seen against a background of relatively lower luminance. Secondly, check A is seen in relatively bright illumination while check B is seen in relatively dim illumination, with an identifiable shadow separating image regions in relatively bright and dim illumination. This contextual difference induces the perceptual effect known as *discounting the illuminant*, whereby check B (and check D) in dim illumination is perceptually shifted in gray shade in order to compensate for the perceived illumination difference. This shift ensures that check B appears similar in gray shade to check C in bright illumination and that check D in dim illumination appears similar in gray shade to check A in bright illumination. This perceptual outcome is commonly termed *lightness constancy* [25]. The computational processes underlying simultaneous contrast and discounting the illuminant appear to combine to produce the dramatic perceptual difference that characterises Adelson's checkerboard display.

[58] sought to characterise the magnitude of perceptual shifts in variants of the Adelson checkerboard display [46] and a related display introduced by [22] among other displays. These authors had subjects adjust the luminance of a matching region, viewed against a black-and-white background, in order to make ‘brightness’ and ‘lightness’ matches to targets viewed within different versions of the checkerboard and simultaneous contrast displays. Two different stimulus conditions were examined. In the “Paint” conditions, all targets were viewed in the context of surfaces depicted as lying under uniform illumination (*without* a shadow overlay) but against surfaces appearing to have different reflectance (‘paint jobs’). In the “Illumination” conditions, the targets were viewed under different depicted illumination levels (*with* a shadow overlay), seen against surfaces appearing to have the same or similar reflectance. Subjects adjusted the luminance of the matching region such that reference and matching regions either appeared to reflect the same “light intensity” (brightness match) or appeared “as if cut from the same paper” (lightness match). These task instructions had little or no influence in the “Paint” conditions, but had a dramatic influence in the “Illumination” conditions. The magnitude of the perceptual shift in the Adelson checkerboard display, for example, was much greater in the lightness matching task than in the brightness matching task. A key goal of the present study is to develop a model that quantitatively predicts how stimulus- and task-driven constraints control the computational processes that contribute to ‘brightness’ and ‘lightness’ matching behaviour [58].

The Anderson-Winawer effect (Fig. 1B)—in which physically identical textured surfaces are seen as either uniform black or white surfaces—depending on the surrounding context, has been theoretically analysed [12], [13] as a perceptual decomposition, or ‘scission’ [6], [9]–[11], [32], into transparent foreground and opaque background layers. The computational process underlying this decomposition is sensitive to the spatial relationship between the target and background stimuli. Rotating the background textures by 90 degrees with respect to the target region, for example, eliminates the effect. According to [12], [13], the visual system uses the fact that *figural contrast polarity* (black-to-white or white-to-black) is preserved around the entire perimeter of the target region to trigger the perceptual decomposition into surface layers. These authors proposed that, once decomposition is triggered, the visual system uses the surface region that appears in ‘plain view’—that is, appearing without the intervening transparent medium—to compute the gray shade of the farther surface layer that is contained within the perimeter of the target region.

[12], [13] provided lightness matching data to support this proposal and showed that the contribution of perceptual decomposition to the effect was far greater than the contribution attributable to simultaneous contrast. Another key goal of the present study is to demonstrate how the same model used to quantitatively predict the contributions of the computational processes underlying the Adelson checkerboard effect and brightness/lightness matching behaviour can also quantitatively predict the perceptual data on the decomposition and simultaneous contrast effects that contribute to the Anderson-Winawer effect.

### Model overview

Two broad classes of computational processes work together to compute surface gray shades in the model: (A) vector summation of luminance and contrast, and (B) vector decompositions implementing the illuminant- and transmittance-shift processes to produce layered representations in different parts of the image.

### General simulation methods

All software implementing the equations and algorithms defined below was written in MATLAB Version 8.0.0 (R2012b). Stimulus luminance values used in the computer simulations were taken from the published values given in [12], [13] and [58].

### Inputs to the model

In order to apply the model to arbitrary images, it would be necessary to solve the image segmentation problem, which generally involves parsing the retinal image into regions differing in either reflectance, illumination or transmittance [8]–[13], [22], [23], [25], [29], [32], [83]. A segmentation process is required in our model in order to (A) define an image region and its contrast with respect to immediately surrounding regions, and (B) divide the image into different regions upon which vector decomposition processes are differentially applied depending on stimulus- and task-driven constraints.

**Figure 3:**
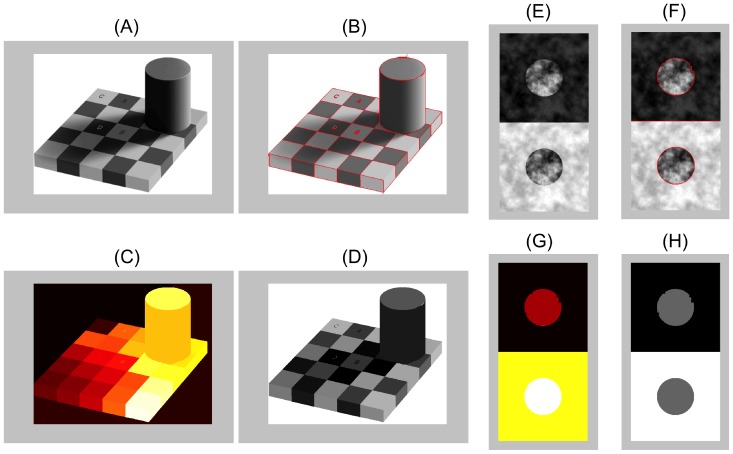
Two examples of image segmentations used to guide the computation of region luminance and contrast. (A) Adelson checkerboard image [1], modified with permission under the Creative Commons Attribution License. (B) Segmentation computed with a standard computer vision algorithm [84] (parameters: 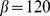, 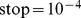). (C) The algorithm returns region labels for each image region. (D) Region labels enable the calculation of mean pixel or luminance values within each segmented region. (E-H) Same as above, except applied to a simple version of the Anderson-Winawer display (adapted from http://www.psy.ritsumei.ac.jp/~akitaoka/AIC2009.html with permission).

Fig. 3 illustrates how a standard segmentation algorithm from the computer vision literature [84] captures the intuition of a suitable segmentation to compute regional luminance and contrast in our analysis. The algorithm segments the Adelson checkerboard image and a simplified version of the Anderson-Winawer display into labelled regions in which mean pixel or luminance values are calculated. The segmented regions are thus characterised by differences in mean luminance, and each individual region is immediately surrounded by one or more regions containing a different mean luminance value.

In the present article, we adopt the following simplifying heuristic to extract predictions from the model. We assume that each check in the Adelson checkerboard image and each target region in the Anderson-Winawer display has been segmented into labelled regions whose mean luminance (more precisely, mean log luminance) we explicitly calculate based on stimulus specifications reported in relevant publications. This allows us to compute the luminance and contrast terms in the model equations, as described in detail below.

The segmentation algorithm can also sometimes produce region labels corresponding to different illumination and transmittance levels (e.g. the border between moons and surrounds in Fig. 3E)—particularly when the regional borders have high contrast—but such regional segmentations are often not computed (e.g. the shadow border in Fig. 3A). We thus explicitly set the values of the free parameters controlling the illuminant- and transmittance-shift processes in a manner consistent with the stimulus-driven constraints (e.g. assuming the same or different illumination levels in different segmented regions), in addition to task-driven constraints (e.g. brightness or lightness matching tasks). In this way, we are able to extract predictions from the model without having to explicitly segment the image into regions differing in illumination or transmittance levels. We are currently developing a version of the model that will incorporate a sophisticated user-guided segmentation process to define regions differing in illumination and transmittance levels in a more general way.

**Figure 4:**
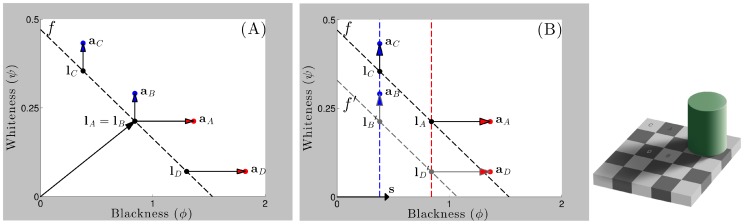
Adelson checkerboard display parsed in the brightness and lightness modes. The model explains the key perceptual properties implied by the Adelson checkerboard display shown in Fig. 1A. Surface gray shades are specified in a perceptual blackness-whiteness space given by the coordinates 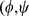). The free parameter 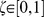 controls the balance between so-called brightness (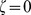) and lightness (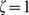) ‘modes’ that represent the respective assumptions of spatially uniform or variable illumination. (A) **Brightness mode**: According to the model, the summation of luminance and contrast vectors ensures that check B in the Adelson checkerboard display has higher whiteness than check A (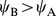 with respect to 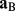 and 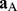) and check A has higher blackness than check B (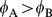 with respect to 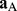 and 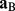), consistent with various data on the simultaneous contrast effect [54]. (B) **Lightness mode**: According to the model, an illuminant-shift process combines with the vector summation underlying simultaneous contrast to produce the Adelson checkerboard effect, i.e. 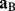 = 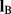 + 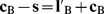, where 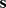 is a ‘shadow vector’ with non-zero blackness and zero whiteness components that introduces the comparison luminance gamut, 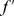. The illuminant-shift process transforms the blackness coordinates of checks B and D in relatively dim illumination towards the blackness axis, e.g. 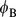 is smaller in lightness mode than it is in the brightness mode example illustrated in subfigure (A). Checks with the same reflectance thus share the same blackness coordinates (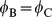), and checks with different reflectance but the same luminance have very different blackness coordinates (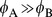 with respect to 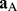 and 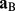). Due to the asymmetrical scaling of blackness coordinates relative to whiteness coordinates, blackness plays the dominant role in determining the surface gray shade [54]. The model thus explains both the independence of surface gray shades with respect to variable illumination intensity levels and the large magnitude of the Adelson checkerboard effect relative to simultaneous contrast alone. Adelson checkerboard image adapted from http://web.mit.edu/persci/people/adelson/checkershadow_illusion.html under the Creative Commons Attribution License.

In our analysis of the Adelson checkerboard (Fig. 4) and the related paint/transparency/shadow display of [58], we shall employ the following notation in order to define contrast in the equations below: A target check in relatively bright illumination will be labelled 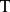 for ‘target’ and surrounding checks of lower or higher luminance than the target will be labelled 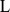 for ‘lower’ or 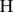 for ‘higher’, respectively. The inputs to the model will then be luminance values labelled either 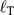, 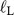 or 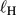. With reference to Fig. 1A, we explicitly define 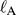, 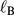, 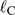 and 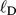 as the luminance values of checks 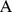, 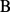, 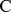 and 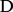, respectively. Thus, when 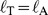, then 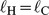 and 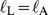 (ensuring that the ratio 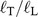 is unity and hence the log of this ratio is zero). Analogous specifications are applied to checks B, C and D in Fig. 1A. When the surround of target 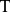 has components that are both lower and higher in luminance than 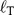 (e.g. a gray target seen against a black-and-white checkerboard, such as the test displays in [12], [13] and [58]) then the ratios of 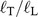 and 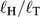 will both be positive.

In our analysis of the Anderson-Winawer display (Fig. 1B), individual pixels within regions 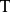, 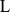 and 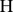 are indexed 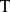, 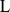 and 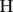, giving luminance values 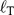, 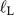 and 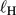, respectively. We then define 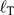, 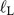 or 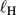 as the geometric mean luminance value of each region (e.g. 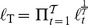), where 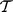 denotes the number of pixels in region 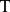. This choice is justified by the fact that these displays are characterised by luminance gradients, meaning that some method of averaging is required to compute contrast. Our choice of the geometric mean luminance is consistent with the logarithmic transformation applied in our model. In the case of the Adelson checkerboard (Fig. 1A), it is the case that 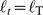, 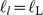 and 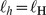. For greatest generality, we write the model equations in terms of these individually indexed luminance values. In general, therefore, we write the luminance of pixel 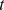 in region 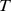 as 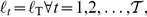
(1)


where for reasons explained below, we label pixel indices in a sequential manner such that 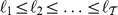.

### Outputs of the model

We now describe the computational model itself, which specifies the algorithmic mapping of image luminance values specified at the pixel level into vector-valued surface representations characterised by ‘blackness’ (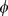) and ‘whiteness’ (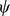) coordinates. In particular, the model maps scalar-valued image representations into vector-valued surface representations. A vector decomposition process produces surface representations that are used to predict human behavioural performance under various stimulus- and task-driven constraints. The output of the model is the vector-valued surface representation, given for each pixel 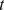 by the equation 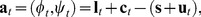
(2)


where the vector components are defined below.

Note that, although model outputs can be displayed as image pairs (i.e. corresponding to 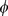 and 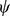 coordinates), we find that displaying outputs in blackness-whiteness coordinate space (e.g. Fig. 4) at selected pixels 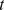 provides greater insight into the model computations. We therefore eschew the common practice of displaying model outputs as images, while still acknowledging that such representations can be useful in certain contexts.

### Model equations

The various vectors comprising Eqn. (2) are defined as follows.

**Figure 5:**
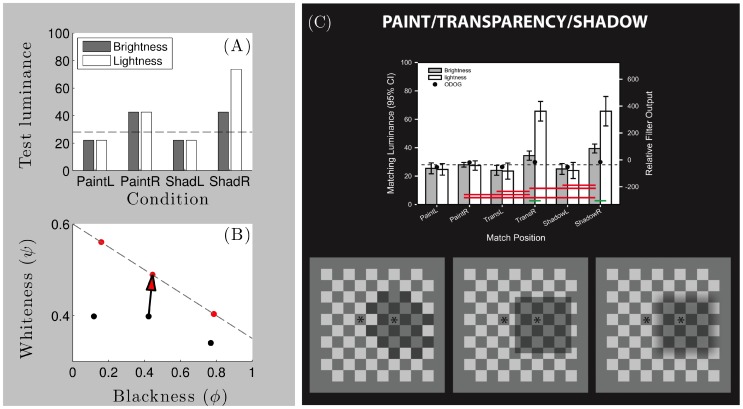
Model predictions of brightness and lightness matching data relating to the Blakeslee-McCourt paint/transparency/shadow display. (A) The model correctly predicts the influence of task instructions on perceptual matches made with surfaces seen under depicted uniform or variable illumination. The luminance of the target is shown by the dashed line, and predictions of luminance of the test target in each condition shown by the level of each bar. (B) Model luminance predictions shown in (A) were generated from minimal Euclidean distances between points representing the reference gray shades (black points, obtained from Eqn. (2) with 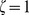 and 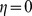) and gamut lines representing the test display (red points on gray dotted line). The test display was assumed to have background luminance values equal to 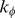 and 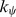, and thus all grey shades in the test display fall on mixed gamut lines, since both blackness and whiteness coordinates have non-zero contrast components. (C) Data and depiction of stimuli reprinted from [58]. In total, there are 12 different test conditions: 6 of these are brightness tasks and 6 are lightness tasks. In (B), black dots indicate the blackness-whiteness coordinates, 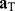, for 8 of these 12 conditions. As the model predictions for the ShadowL and ShadowR conditions are equally applicable to the experimental TransL and TransR (transparency) conditions, we omit the 4 transparency conditions. There are only 3 unique coordinates, since the same blackness-whiteness coordinates at approximately 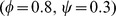 are obtained for all L conditions, and the same coordinates at approximately 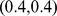 are obtained for both PaintR conditions and ShadowR (labelled ShadR above) in the brightness task. The final black dot at approximately 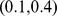 occurs uniquely for ShadowR in the lightness matching task. The red arrow indicates the minimal perceptual match between reference and test coordinates for both PaintR conditions and ShadowR in the brightness matching task.

- A *luminance vector* is given by

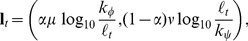
(3)where 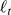 is defined above, 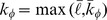 and 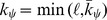 are ‘anchoring’ parameters, 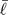 is the highest luminance value in the entire display, 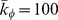 and 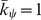 are constants, and 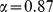, 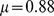 and 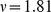 are estimated constants based on psychophysical data [54]. We term the blackness and whiteness components of the luminance vector *luminance blackness* and *luminance whiteness*, respectively.
  The anchoring scheme defined above implies that scenes with luminance values below 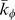 will contain no white surfaces, but scenes with luminance values above this threshold will contain one white surface corresponding to the highest luminance value in the scene. We have found that this rule—coupled with our choice of value for 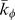 and 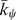
—is suitable to model the perception of diffusely reflecting surfaces rendered on low dynamic range displays viewed under typical daylight adaptation conditions. See [56] and [52] for discussions of more complex anchoring rules in the context of brightness, lightness and gloss perception.
- A *contrast vector* is given by

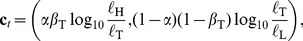
(4)where 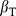 represents the proportion of the surrounding region with luminance higher than the target, as in the equation 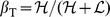. In practice, we set 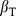 by hand in a manner consistent with this equation. We refer to the blackness and whiteness components of the vector specified in Eqn. (4) as *contrast blackness* and *contrast whiteness*, respectively. Note that the individual scalar components defining the contrast and luminance vectors above are summed to give the values of 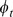 and 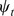 defined in Eqn. (2).
- An *illuminant-shift* vector (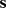) specifies the magnitude of ‘illuminant-discounting’ in a manner that depends on the ratio of the highest-luminance regions designated as appearing in relatively bright illumination (labelled 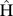) and dim illumination (labelled 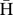), respectively. The illuminant-shift vector is expressed as

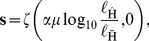
(5)where 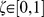 is a free parameter representing various stimulus- and task-driven constraints [52], as discussed below. In the perceptual demonstrations of surface and shadow perception analysed in this article (Fig. 1A, Fig. 5B), the illuminant-shift vector is applied asymmetrically; namely, only to those target regions in dim illumination (e.g. checks 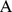, 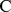 in Fig. 1A), not bright illumination (e.g. checks 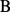, 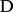 in Fig. 1A). The illuminant-shift process constitutes a mathematical decomposition of the vector 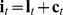 into surface 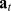 and shadow 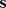 component vectors, such that 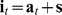.
- A *transmittance-shift* vector (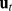) specifies the magnitude of ‘transmittance-discounting’ relative to pixels designated as appearing in ‘plain view’

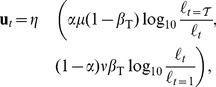
(6)where 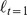 and 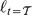 equal the lowest and highest luminance values within the target region, respectively, and 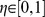 is a free parameter representing figural-continuity (i.e. spatial continuity of contours across figure and ground regions) and contrast-polarity (i.e. continuity of border polarity between figure and ground regions) constraints that are known to characterise scission into transparent layers [6], [8]–[13], [85]. Note that 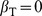 and 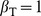 when the surround has higher and lower geometric mean luminance than the target region in the Anderson-Winawer display (Fig. 1B), respectively. By Eqn. (6), then, whiteness coordinates are shifted when the target is a decrement and blackness coordinates are shifted when the target is an increment, which is what is required to discount the physical transmittance shift in a manner consistent with figural contrast polarity (see Results). The transmittance-shift process defined above is proposed to underlie the separation of figural regions into figure and ground layers in a manner consistent with the figural-continuity and contrast-polarity constraints reported in extant perceptual studies [12], [13], [40]. Indeed, the transmittance-shift process with 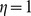 constitutes a mathematical decomposition of the vector 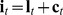 into figure 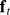 and ground 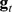 component vectors, such that 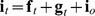, where 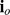 is the origin of the vector decomposition.

### Model parameters and properties

**Figure 6:**
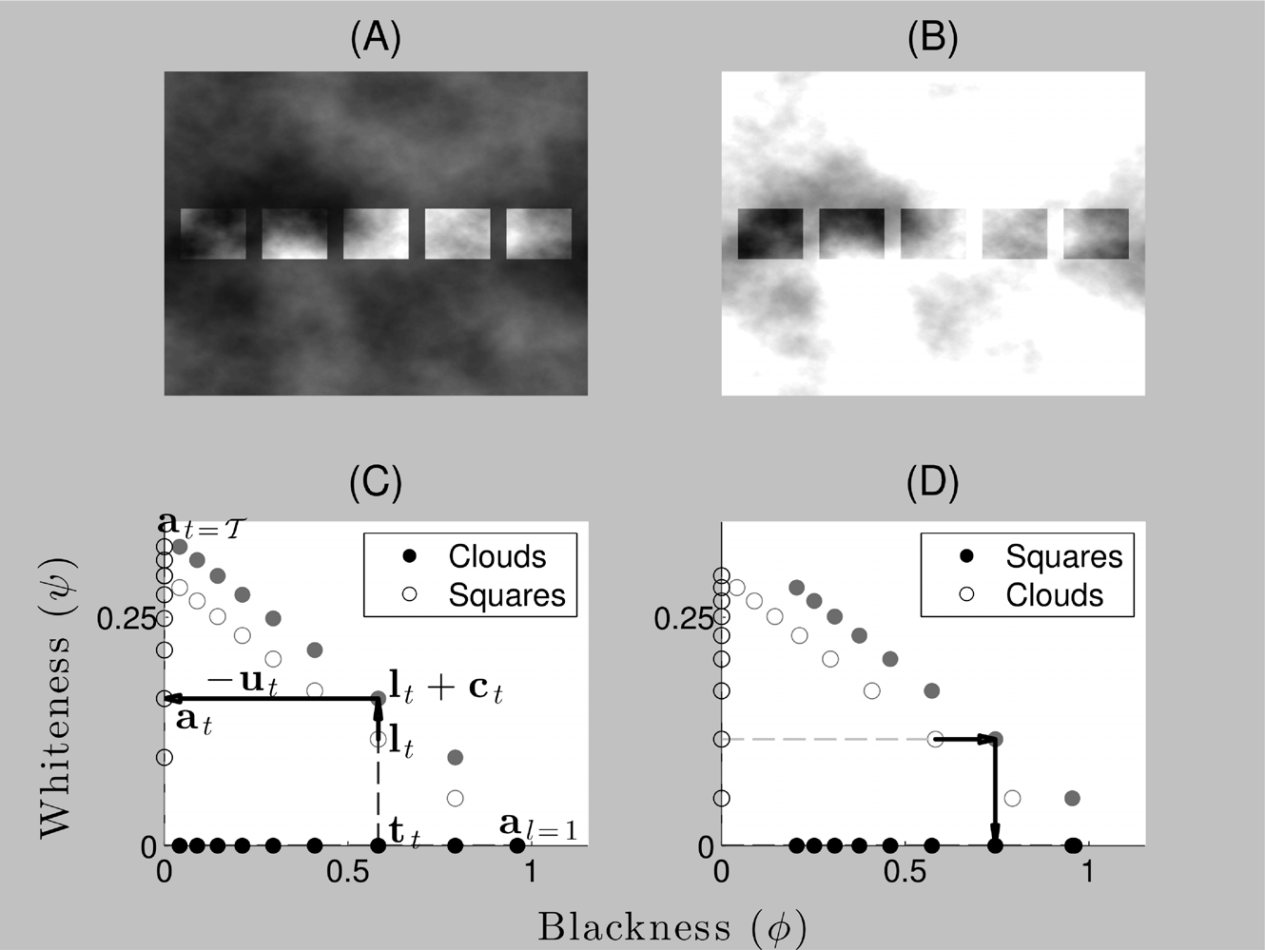
Anderson-Winawer display parsed in the brightness and lightness modes. (A,B) The Anderson-Winawer display with blackish and whitish backgrounds, respectively. (C,D) **Brightness mode**: The empty gray circles (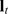 with 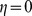) form the standard luminance gamut line for each pixel contained within each of the whitish or blackish squares shown in (A,B). The filled gray circles (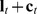 with 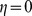) form the standard increment and decrement gamut lines in (C) and (D), respectively, similar to Fig. 4A. These points, which are offset from the standard luminance gamut due to addition of the whiteness and blackness contrast vectors, would correspond to the perceived gray shades in (A,B) if the squares where rotated by 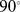 (rotation now shown here). **Lightness mode**: The model explains how the visual system computes separable whitish and blackish figural surface layers (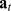) through blackish and whitish transparent ‘ground’ layers (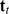) when 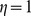. The transmittance-shift process subtracts the vector 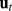 from each filled gray circle to compute each 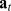 (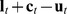 with 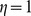). Surface layers are composed of the collection of every 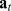, represented here by the empty and filled black circles falling on the whiteness and blackness axes, respectively. The vertical and horizontal rows of empty and filled black circles thus correspond to the perceptually whitish and blackish layers evident in (A,B), respectively. The labelled vector corresponds to 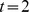, 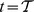 denotes the whitest pixel within the target region, and 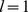 denotes the blackest pixel in the surrounding region. Note that 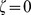.

We now highlight some key conceptual properties of the model, some of which have previously been detailed in recent publications [52], [54]–[57]:

- We assume in what follows that 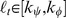. This assumption implies that the blackness and whiteness components of the luminance vector in Eqn. (3) are always non-negative. Likewise, the blackness and whiteness components of the contrast vector in Eqn. (4) are by the definitions of 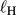 and 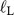 also always non-negative. These constraints thereby ensure that the blackness and whiteness components of the sum of luminance and contrast vectors are always non-negative. This is why all points shown in Fig. 4A, for example, are restricted to the upper right quadrant of blackness-whiteness space.
- Assume 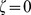 in Eqn. (5) and 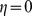 in Eqn. (6). Now consider an image with uniform luminance, 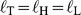 (i.e. a Ganzfeld [62]). All pixel indices 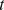 in the image will have zero contrast values in both the blackness and whiteness coordinates. The blackness coordinate is then zero when 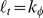 and the whiteness coordinate is zero when 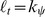. We write the corresponding whiteness and blackness coordinates to these two cases as 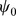 and 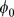. The *standard luminance gamut* is then defined as all points on a negatively sloped straight line in blackness-whiteness space defined between these two axis intercepts, 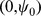 and 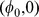. This can be expressed as the equation

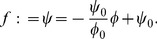
(7)All luminance vectors, 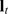, are constrained to fall on the standard luminance gamut line, 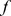; that is, letting 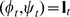 satisfies Eqn. (7). The black dotted lines in Fig. 4A,B, for example, represent the standard luminance gamut.
- In the case of a simple image with a single uniform target region on a *uniform* background region 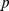, the blackness-whiteness coordinates corresponding to a pixel 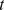 within the target region will not fall on the standard luminance gamut, due to the contrast terms in the blackness-whiteness equations. As can be seen from Eqn. (2), the deviation from the standard luminance gamut is given by the contrast vector, 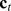. In the case of a contrast increment, the term 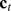 will have a non-zero whiteness co-ordinate and a zero blackness co-ordinate. This contrast whiteness component is added to the luminance vector to define a *standard increment gamut*. In the case of a contrast decrement, the term 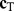 will have a non-zero blackness co-ordinate and a zero whiteness co-ordinate. This contrast blackness component is added to the luminance vector to define a *standard decrement gamut*. For images containing both contrast increments and decrements (e.g. a checkerboard pattern), both contrast components will be non-negative. The contrast vector will then add both blackness and whiteness components to the luminance vector, defining a *standard mixed gamut*. In fact, in general it is possible to define families of gamut lines, both standard and comparison, each depending on stimulus- and task-related factors (see [52], [55], [56] for further details). One could, for example, draw separate comparison increment and decrement gamut lines through each individual blue and red point shown in Fig. 4B, but we shall omit these lines in order to maintain figural clarity.
- Now consider an image within which pixels indexed by 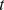 are identified as appearing in relatively dim illumination. In this case, 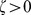 and 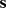 becomes non-zero; a new gamut line representing surfaces appearing in the relatively dimmer illumination level is thus defined. We introduce 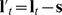, such that the blackness-whiteness coordinate of pixel 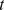 is 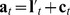. We then define a *comparison luminance gamut* as

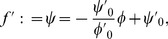
(8)which has a smaller whiteness intercept than the standard luminance gamut (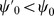), indicating a relatively lower illumination level, but lies parallel to the standard luminance gamut, such that 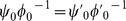. All lum inance vectors, 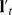, are constrained to fall on the comparison luminance gamut, 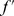; that is, letting 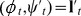 satisfies Eqn. (8). The light gray dotted line in Fig. 4B, for example, represents the comparison luminance gamut when 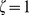. According to the equation 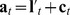, then, it is further possible to define increment, decrement and mixed *comparison* gamuts. For detailed discussion of the computational utility of the relationship between standard and comparison gamuts, see [52], [55], [56].
- Perceptual matches performed in psychophysical experiments generally correspond to *minimal perceptual mismatches* between points specified to lie along different gamut lines [52]–[54]. The minimum perceptual distance between a reference point lying on a gamut line specified for a reference display and the set of all points on another gamut line specified for the test (or matching) display determines the predicted luminance setting. It is calculated as the luminance value that minimises the Euclidean metric, 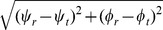, where indices 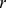 and 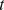 denote reference and test targets, subject to the constraints imposed by the test gamut line. The blackness-whiteness plot shown in Fig. 5B provides an example of the manner in which the idea of minimal perceptual mismatches can help to account for perceptual data. Previous theoretical and experimental work also supports the idea that subjects’ cannot generally make satisfactory brightness matches between targets viewed against backgrounds differing in luminance or perceived illumination level [52]–[54].
- The parameter 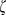 controls the balance between two perceptual ‘modes’ that each explain key properties of brightness and lightness perception, respectively (the parameter 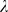 in [52] is equivalent to 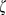 here). Under the assumption that luminance variations between pixels are due entirely to reflectance variations, blackness coordinates are primarily correlated with local luminance (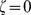; brightness mode). Under the assumption that luminance variations between pixels are due entirely to illumination variations, blackness coordinates are primarily correlated with diffuse surface reflectance (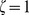; lightness mode). Intermediate values of 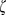 represent a ‘balance of probability’ [86], [87] between these two extreme assumptions and thus represent linear combinations of presumptive illumination and reflectance variations. Here we generalise the distinction between brightness and lightness to describe the surface perception under the assumption that 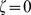 and 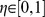; that is, by generalising the definition to the case of surface perception through transmissive media (e.g. Fig. 6).
- The parameter 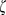 is itself a function of both the stimulus (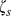) and task (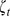), such that 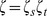, where 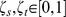. The assumption of uniform illumination corresponds to 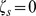 (e.g. the “Paint” condition of [58]). The assumption of variable illumination corresponds to 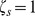 (e.g. the “Illumination” condition of [58]). As 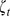 can only modify the value of 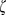 when 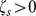, this construction is consistent with psychophysical data reported in [58] showing that task-driven constraints on matching behaviour can only exert an influence when stimulus conditions support the perception of variable illumination. In the “Lightness” matching task of the “Illumination” conditions in [58], we assume that 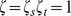, whereas in the “Brightness” matching task we assume that 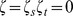. This construction reflects the fact that, under the assumption of uniform illumination across a scene, luminance and surface reflectance are correlated, whereas under the assumption of variable illumination, luminance and reflectance are uncorrelated. The capacity to flexibly switch between perceptual modes correlated with either luminance or reflectance thus underscores a key conceptual deviation of our model from the classical theory of surface perception as a problem of reflectance recovery.
- Blackness-whiteness space is asymmetrically scaled, meaning that a unit variation in physical luminance maps to a far greater variation in blackness coordinates than whiteness coordinates [52], [54]. This proposal explains a wide range of otherwise puzzling data concerning asymmetries in the perception of contrast increments and decrements. The asymmetry can be appreciated, for example, by comparing the scales of the 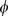
- and 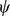-axes in Fig. 4. The precise ratio of blackness/whiteness variation depends on various factors, but has been estimated to be no less than approximately 3 [54]. Given the setting 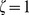 and 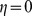, then under the assumption that surfaces seen under different illumination levels contain identically distributed sets of reflectance values, pairs of points associated with 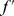 and 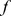 that have the same blackness coordinates (e.g. 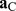 and 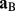 in Fig. 4B) are perceptually more similar to one another than pairs of points associated with 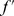 and 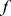 that have different blackness coordinates (e.g. 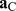 and 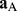 in Fig. 4B) [52].

## Results

### Surface perception under uniform and variable illumination

We now show how our model accounts for key properties of surface perception under uniform and variable illumination in the Adelson checkerboard effect. We claim that the effect actually consists of two distinct effects: simultaneous contrast and illuminant discounting. We first briefly recapitulate our previously published account of simultaneous contrast [54] in terms of the Adelson checkerboard display (Fig. 4A).

Our explanation of simultaneous contrast is most easily understood by assuming that the Adelson checkerboard display is parsed by the visual system such that only a single illumination level is perceived (i.e. by assuming that 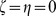). In other words, the ‘shadow’ region is actually perceived as having relatively lower reflectance than the ‘brightly illuminated’ region. As indicated above, the parameter setting of 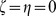 represents the brightness mode in gamut relativity. The luminance vector associated with each check (e.g. 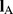 and 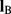, where subscripts are used as labels rather than indices) are all constrained to fall on the standard luminance gamut, as defined by Eqn. (7), which is represented by the black dotted line in Fig. 4A. Checks with the same luminance (i.e. checks A and B) are thus mapped to identical points on the standard luminance gamut (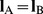). The points 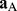, 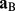, 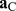 and 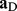 in Fig. 4A represent the blackness-whiteness coordinates of checks A, B, C and D following the addition of the contrast vector to the luminance vector (e.g. 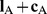). The coordinates 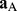 and 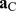 thus diverge, with a contrast blackness component added to 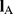, which is surrounded by brighter checks (check A) and a contrast whiteness component added to 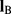, which is surrounded by darker checks (check D). Checks A and B are thus mapped to blackness-whiteness coordinates that correspond to two different gray shades, 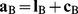 and 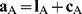.

Gamut relativity predicts that check B will be perceived as both ‘blacker’ and ‘less white’ than check A since the blackness coordinate of check A is larger than that of check B and the whiteness coordinate of check B is larger than that of check A. This prediction is generically consistent with the occurrence of the simultaneous contrast effect. As discussed in [54], moreover, this account of simultaneous contrast is quantitatively consistent with ‘brightness matching’ data and explains the inability of subject's to make satisfactory brightness matches when reference and test targets are viewed against backgrounds differing in luminance.

Our explanation of the large perceptual shift evident in the Adelson checkerboard display assumes that the display is parsed by the visual system into two different illumination levels (i.e. by assuming that 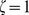). Fig. 4B illustrates the model account of the appearance of the Adelson checkerboard display when the illuminant-shift process is engaged. Given a parameter setting that represents the lightness mode in gamut relativity (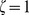), the perceived difference in illumination level over the display is represented in the fact that 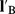 and 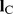 now fall on separate luminance gamut lines, 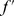 and 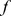, respectively. Due to the process of discounting the illuminant, the blackness coordinates (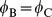, 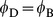) of the vector pairs 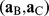 and 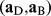 remain invariant to differences in the depicted illumination intensity across the display. The perceptual shift between 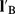 and 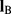 is equal in magnitude but opposite in sign to the physical shift in blackness induced by the illumination difference. The shift is given by the vector, 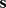, which specifies the magnitude of the discounting according to Eqn. (5), under the assumption that 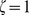. The shifted luminance vector coordinates are added to the contrast vectors to give 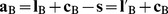. As the coordinates of check A in bright illumination remain unaffected by the discounting process, the magnitude of the difference between the vector 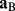 and 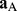 is much greater than the magnitude of the difference between the untransformed vectors in the brightness mode, given by 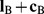 and 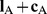 (Fig. 4A). The Adelson checkerboard display thus induces a far larger perceptual shift than would be expected on the basis of the processes underlying simultaneous contrast alone.

This perceptual shift can be understood as a manifestation of computational processes operating with the goal of parsing the retinal image into distinct surface and shadow layers. This goal can be clarified by first rewriting the equation 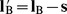 in the form 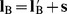. This equation says that the standard luminance vector associated with check B (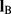) is equal to the comparison luminance vector (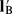) plus the shadow vector (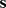). In other words, the illuminant-shift process decomposes the standard luminance vector into surface and shadow vectors whose sum equals the original standard luminance vector. Due to this decomposition, 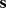 falls on the blackness axis and 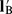 has been shifted towards the whiteness axis by the amount 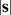. The decomposition thereby gives rise to the following property: The distance between the 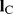 and 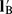 is less than than the distance between 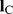 and 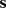; that is, the inequality 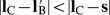 holds, given that 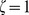 and 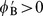. We claim that this inequality provides the basis for the capacity of the visual system to parse the Adelson checkerboard display into surface and shadow ‘layers’. It ensures that points in backness-whiteness space representing physical surfaces in dim illumination can be unambiguously ‘assigned’ to corresponding points in bright illumination; that is, without interference from points representing shadows, which have been ‘displaced’ onto the blackness axis. These model properties thus explain the emergence of layered perceptual representations corresponding to surfaces and shadows.

To further emphasise the unique features of our model, we now analyse how the visual system might flexibly switch between brightness and lightness modes based on stimulus- and task-specific constraints. In this respect, we analyse data pertaining to the paint/transparency/shadow display used in [58]. In particular, we attempt to predict how stimulus- and task-driven constraints interact to determine brightness and lightness matches when the display appears either uniformly or variably illuminated (i.e. paint *versus* shadow, though the model predictions for the shadow condition apply equally well to the transparency condition). The model predictions are shown in Fig. 5A alongside the psychophysical data in Fig. 5C, and agree reasonably well with the data. The model predicts the data well, with the main discrepancy being that the model predicts a slightly too strong simultaneous contrast effect with increments under uniform depicted illumination (c.f. condition PaintR) than is observed in the data. Of particular importance is to note that the model correctly predicts that lightness matching instructions have a disproportionately greater influence on contrast increments relative to decrements. This is because the model predicts that the increment region, which appears in dim illumination, undergoes the discounting, rather than the decrement region, which appears in bright illumination. Concordantly, the matching instructions have little influence in the latter case, but a large influence in the former case (i.e. condition ShadowR).

As discussed above, brightness and lightness matches are understood in the model as minimal perceptual mismatches between points lying on different gamut lines. The red test (or match) points lying on the dotted gray gamut line in Fig. 5B, for example, represent gray shades that minimal Euclidean distances with respect to the black points representing the gray shades of the target reference surfaces lying on different gamut lines (not shown). A key prediction of gamut relativity that sets it apart from alternative models [58] is thus that subjects cannot generally make satisfactory brightness or lightness matches [52]–[54]. Indeed, the model makes precise quantitative predictions that can be suitably compared against perceptual data obtained under conditions where subjects rate the perceptual similarity of their own matches [52]–[54], [88]. The model is also consistent with perceptual data indicating that distinct computational processes subserve discrimination of targets against their local backgrounds (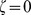) and lightness matching performance (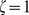) [46].

### Surface perception through atmospheric media

We now show how our model generalises to naturally account for properties of figure-ground separation and surface appearance through atmospheric media in terms of the Anderson-Winawer effect. We begin by illustrating the summation of luminance and contrast vectors in the brightness mode with 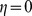 (Fig. 6C,D). In the absence of scission cues (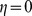), a single figural surface layer appears in plain view and is thus described as a surface brightness layer, according to the definition provided above. These vectors are given by the equation 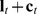. This latter situation occurs, for example, when the background regions of the Anderson-Winawer display are rotated by 90 degrees with respect to the target regions. The unfilled gray points shown in the blackness-whiteness plots of Fig. 6C,D correspond to a selection of pixels from within the square parts of the displays shown in Fig. 6A,B, and illustrate a mapping of physical luminance to standard luminance gamut. Note that these points are the same in Fig. 6C,D, since the physical luminance of all points in the squares in Fig. 6A,B are identical. The contrast vector 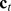 is associated with a pure whiteness ‘boost’ for figural contrast increments and a pure blackness ‘boost’ for figural contrast decrements. These contrast components are depicted as vertically and horizontally oriented whiteness and blackness vectors adding to the luminance vectors in Fig. 6C,D. For figural contrast increments (Fig. 6C), for instance, the boost shifts points on the standard luminance gamut upwards to form the standard increment gamut.

Given strong cues to the presence of transmissive media in an image, we assume that 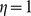. The model equations then allow us to define 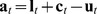 to represent the underlying figural surface layer. The parameter 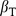 determines the orientation of the vector 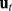; it is horizontal for target increments and vertical for target decrements. We may thus define a vector orthogonal to 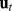 and with different length using the definition 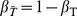; that is, we define the vector 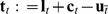 to represent the transparent layer ‘belonging’ to the ground region surrounding the figure region. The transmittance-shift vectors 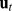 and 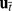 thus operate on each 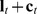 to compute each 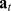 and 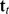. The shifts introduced by these vectors are equal in magnitude but opposite in sign to the physical shifts in blackness and whiteness induced by the transmittance difference between the ground medium and the underlying figural surface region seen in plain view (defined as 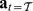 in Fig. 6C). The application of these vectors implies that either blackness or whiteness coordinates always remains constant with respect to differences in the physical transmittance of the ground medium. This ensures, for example, that each 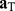 in Fig. 6C always lies closer to 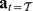 than does 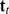; that is, the inequality 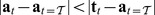 holds for any 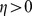.

This invariance is proposed to underlie the ‘grouping’ of vectors into perceptual layers characterising physically transmissive filters and media [55]. The net effect is to discount the transmittance of the ground medium in computing the underlying figural surface layer. The model thereby separates the figural image region into figure and ground layers, thereby accomplishing figure-ground separation. As indicated in the Model section, the transmittance-shift process with 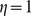 is mathematically identical to a vector decomposition of the vector 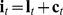 into figure 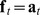 and ground 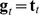 vectors corresponding perceptually to the figure and ground layers within the figural region.

In the Anderson-Winawer effect, this computational process generates the perceptual difference engendered by varying the mean luminance of the ground region outside the figure. In the case of the blackish ground region, vector decomposition operates to transform points on the standard increment gamut into a column of points lined up on the vertical constraint line provided by the whitest pixel of the underlying surface that appears in plain view. In the case of the whitish ground region, vector decomposition operates to transform points on the standard decrement gamut into a row of points lined up on the horizontal constraint line provided by the blackest pixel of the ground region that appears in plain view. The net effect is to produce separate sets of vectors corresponding to the ground and figural surface layers. As discussed above, each surface vector has the property that it lies closer to the surface vector appearing in plain view than does its ‘partner’ ground vector, allowing individual surface vectors to group together to form Gestalt-like representations of surface gray shades [25]. For figural contrast increments, for example, the vertical column of vectors lying on the whiteness axis form a whitish underlying figural surface layer by virtue of their relationship to the whitest figural pixel in plain view.

**Figure 7:**
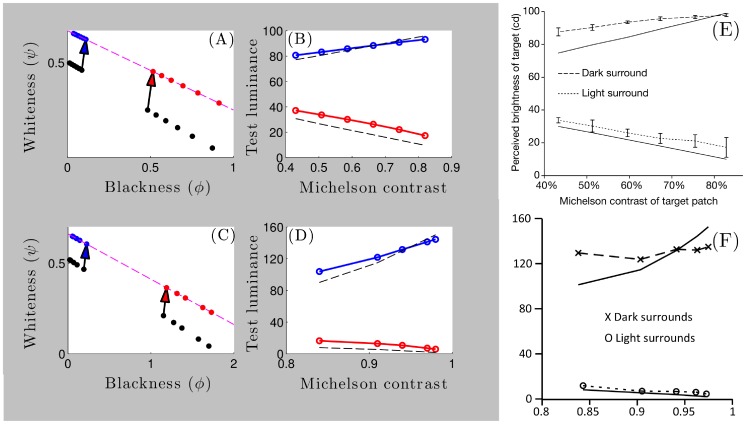
Model predictions of lightness matching data relating to the Anderson-Winawer effect. (A,C) Model luminance predictions were generated from minimal Euclidean distances between points representing the reference gray shades (black points) and gamut lines representing the test displays (red/blue points on purple dotted lines). Each black dot represents either the highest or lowest luminance value within the target reference region associated with each Michelson contrast level, depending on the figural contrast polarity of the reference region (i.e. highest for black dots matched to blue dots, and lowest for black dots matched to red dots) (B,D) The model correctly predicts that subjects set luminance values higher than the luminance values of the target reference region appearing in plain view (black dotted lines) for both figural contrast increments (blue points/lines) and decrements (red points/lines). Each empty blue and red dot corresponds to one of the filled blue or red dots in (A,C). Higher luminance values map to higher whiteness values and lower blackness values, respectively. (E) Data reprinted from [12]. (F) Data replotted from [13].

To quantitatively assess the predictions of the model with respect to perceptual data, we calculated predictions of ‘lightness matches’ (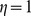) for various Michelson contrast values of the target regions in the Anderson-Winawer display (Fig. 7A,C), as reported in [12], [13]. Fig. 7B,D shows the model predictions alongside the perceptual data in Fig. 7E,F. The model correctly predicts that subjects’ luminance settings always lie above the line indicating the luminance of the whitest or blackest pixels associated with figural contrast increments and decrements, respectively. This bias is a direct consequence of the asymmetric scaling of blackness-whiteness space, which forms a key computational feature of gamut relativity. In particular, the dominance of blackness with respect to whiteness ensures that the model weights the contrast blackness component more strongly than the contrast whiteness component. This causes a nominal gray shade seen against a black/white checkerboard, or black/white noise image, as used in the test displays reported in [12], [13], to appear relatively whiter and less black than the reference region seen against more neutral backgrounds. The model thus compensates for this bias by selecting luminance values higher than those associated with pixels in plain view to produce the best ‘lightness match’. Perceptual data on the Anderson-Winawer effect thus supports many of the key modelling postulates underlying gamut relativity. We leave to future work the goal of determining whether the model can accurately predict surface perception in the presence of simultaneous variations in both illumination and transmittance levels (i.e. with both 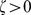 and 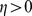 [18]).

## Discussion

We have presented a model that quantitatively accounts for perceptual data relating to some of the most striking and theoretically important effects of layered perceptual representation and surface appearance reported in the literature. In particular, the model reported in this paper documents four (4) key advances with respect to previously published work. The model: (1) provides the first unified analysis of how the visual system represents surfaces independently of shadows and atmospheric media, as exemplified in the Adelson checkerboard and Anderson-Winawer effects; (2) reconciles and unifies two prominent theories of surface lightness; (3) quantitatively predicts how stimulus- and task-driven factors combine to control brightness/lightness matching behaviours reported in published perceptual experiments; (4) unifies two previously published gamut relativity models, aimed at explaining properties of brightness/lightness perception [52], [54], lightness/transparency perception [55] and lightness/gloss perception [56], respectively. The model thus provides the first unified account of the mid-level computations underlying layered perceptual representation, which are believed to subserve the high-level computations involved in the identification of surface materials [1], [2].

As indicated above, the model unifies two prominent theoretical approaches to surface lightness, known as the ‘anchoring’ and ‘scission’ theories [8]–[14], [22]–[25], [29], [31]–[34], which have previously been applied separately to study the types of effects illustrated in Fig. 1. Lightness anchoring theory [25] posits that the visual system parses the scene into differentially illuminated regions, as in gamut relativity, before mapping relative reflectance values within each illumination level to absolute surface lightness values. This computation is captured in the current model in terms of the ‘illuminant-shift’ process applied to the blackness dimension. This process also generates a representation of the shadow layer. Scission theory [8]–[14], [31]–[34] posits that the visual system parses the scene into layered representations, one seen through another, in order to disentangle the differential effects of surface reflectance and atmospheric media. This is accomplished by first estimating which surface regions appear in ‘plain view’ and which surface regions appear through atmospheric media of variable physical transmittance [12], [13]. This computation is captured in the current model in terms of the ‘transmittance-shift’ process that is applied either to the blackness or whiteness dimensions, depending on figural contrast polarity. The computational outputs of the illuminant- and transmittance-shift processes are then combined in a single equation to compute layered representations. The current model thus mathematically unifies the central concepts in the lightness anchoring and scission theories.

The novel account of brightness and lightness perception embodied in gamut relativity may partially account for the wide range of behaviours observed when subjects perform perceptual matching tasks. At one extreme, task instructions to perform either brightness and lightness matches appear to have little or no influence on perceptual matches in the absence of a visible transparent layer. Such perceptual matches are associated with low intra- and inter-subject variability and tend to be subjectively relatively easy to make [58]. At the other extreme, lightness matches made under conditions where the task is largely underdetermined by stimulus-driven constraints—that is, in the absence of surface regions appearing in plain view—are associated with high intra- and inter-subject variability and tend to be subjectively relatively difficult to make. In such conditions, subjects may adopt a wide range of criteria to perform the matching task, such as attempting to ‘infer’ the surface appearance of the target under a certain illumination level [41]–[45], [48], [49]. In the middle ground, lightness matches made under conditions where stimulus-driven constraints are strongly present—that is, when surface regions appearing in plain view provide strong cue to the magnitude of the illuminant shift in shadow—are also associated with low intra- and inter-subject variability and tend to be subjectively relatively easy to make [58]. It is this class of lightness match that we have focused upon in this article. We expect to generalise our model to the more ‘inferential’ class of lightness match by demonstrating how subjects can ‘infer’ surface appearance under different gamut lines (i.e. by inferring the magnitude of the illumination or transmittance shift). The model thereby promises to provide a unified account of a wide range of matching strategies employed by human subjects in various experimental situations.

In providing a unified and general account of perceptual layering and surface appearance, our model provides crucial insights into the remarkable capacity of the human visual system to identify surface materials, illumination and atmospheric media. One potential application of this modelling framework involves the design of computer graphics software that allows a user to create layered image representations by explicitly controlling perceptual variables (e.g. lightness and transparency), rather than indirectly specifying physical variables in models of light transport (e.g. reflectance and transmittance). We are also developing our modelling framework to leverage user-based image segmentation algorithms in a manner that will allow the user to predict brightness, lightness, transparency and gloss levels from arbitrary images.
